# The Positive Effect of Video-Game Play on College Students’ Anxiety and Depression Symptoms During the COVID-19 Pandemic Shelter-in-Place Lockdowns: Mixed Methods Study

**DOI:** 10.2196/58857

**Published:** 2025-05-30

**Authors:** Fengbin Hu, Zixue Tai, Jianping Liu

**Affiliations:** 1 Research Center for Cyberspace Governance MOE Laboratory for National Development and Intelligent Governance Fudan University Shanghai China; 2 School of Journalism and Media University of Kentucky Lexington, KY United States; 3 School of Journalism and Communication Minjiang University Fuzhou China

**Keywords:** anxiety, depression, video games, online games, mobile games, college students, COVID-19, COVID-19 pandemic lockdowns, China

## Abstract

**Background:**

A growing body of research has examined the connection between video game play and relief from anxiety and depressive symptoms during the COVID-19 pandemic years. However, evidence has been limited in dissecting the role of video games in moderating personal health outcomes such as anxiety and depression. This research aimed to provide insights into this area by investigating Chinese college students living under difficult conditions during the COVID-19 pandemic shelter-in-place lockdowns.

**Objective:**

This study investigated the effect of video games on mental disorders among Chinese college students during the COVID-19 pandemic shelter-in-place lockdown mandates.

**Methods:**

A mixed methods approach was adopted. The quantitative portion included a cross-sectional survey of a national sample of 2818 (n=1396, 49.54% male vs n=1422, 50.46% female) college students from 8 provinces spanning 16 geographic regions during the extended COVID-19 pandemic lockdowns in late 2021 and early 2022. The qualitative portion encompassed 17 semistructured in-depth interviews of (9/17, 53% female vs 8/17, 47% male) students on their experiences, perceptions, and evaluations of playing video games during the lockdowns. Anxiety symptoms were measured using the 7-item self-administered Generalized Anxiety Disorder scale, while depression was assessed with the 9-item Patient Health Questionnaire scale. Multiple regression analyses were used to examine whether demographic variables (ie, sex and year in college), modality and content of play, and play time accounted for the outcomes of anxiety and depression. ANOVA tests were performed on overall playtime and the prelockdown-to-lockdown period change in game time on the severity of anxiety and depression symptoms. Thematic analysis of qualitative data provided additional perspectives on gaming dynamics in relation to anxiety and depression.

**Results:**

No significant sex effect was detected in video game play outcomes for anxiety or depression. At the level of the study population, a significant difference between gamers and nongamers was observed to moderate anxiety (*t*_2816_=−0.42, *P*=.02) but not depression (*t*_2816_=−0.12, *P*=.60) symptoms, controlling for the effect of sex. Playing more online games, spending more time gaming, and an increase in play time were linked to heightened anxiety and depression symptoms. Conversely, playing with friends was connected to lowered anxiety (β=−0.05, *P*=.04) and depression (β=−0.08, *P*=.003) scores.

**Conclusions:**

The buffer effects of video games may be strongest among routine players with moderate to low anxiety and depression symptoms, while excessive gaming, as shown in the overall amount of game time as well as the prelockdown-to-lockdown period increase in game time, may have detrimental consequences among those struggling with high anxiety and depression. Social play was an effective mechanism in mitigating anxiety and depressive tendencies. Future research should address game content and modality as well as the long-term impacts of video game engagement during crises.

## Introduction

### Background

The Global Burden of Diseases, Injuries, and Risk Factors Study has consistently shown that mental disorders are among the leading causes of global health-related burden, with no evidence of reduction since 1990, while the 2019 Global Burden of Diseases, Injuries, and Risk Factors Study showed that anxiety and depressive disorders were the 2 leading mental disorders in the world [1,2]. The World Health Organization, which advocates mental health as a basic human right, noted in its survey of member nations that, despite increasing interest in and understanding of mental health in the past 20 years, mental health care systems and services are ill-equipped in most countries, and nonexistent in some low-income regions, to handle people’s needs [3].

Globally, the university student population is a high-risk demographic in developing mental health problems [4,5], and the number of students with serious emotional and mental health problems has been on the rise across college campuses in the 21st century [6,7]. In particular, there have been substantial increases in the prevalence rates of symptoms of depression, anxiety, and suicidal ideation among college students in recent decades [8-10]. The global crisis created by the COVID-19 pandemic has posed a monumental threat to the physical and mental well-being of the international community, and college students are especially vulnerable to elevated rates of anxiety, depression, and stress during moments of heightened uncertainties [11,12]. Moreover, self-isolation, stay-in-shelter lockdowns, and closures of college campuses have been enforced at times in most countries as an effort to contain the spread of the coronavirus. College students under lockdown reported spikes in stress, anxiety, and depressive symptoms as well as a lack of motivation, restless sleep, and loneliness [13,14]. In China, varying degrees of lockdowns have been implemented, ranging from neighborhood units to cities and whole provinces, at different times based on the severity of local spread of infections as a fundamental national strategy toward a zero-COVID policy [15]. Lockdowns have been found to exacerbate devastating psychological consequences and complicate interventions and coping at both the communal and individual levels [16].

Meanwhile, video games have been a leading sector of global growth and innovation in recent decades and have become a central part of people’s cultural lives [17]. Besides their entertainment value, games have been associated with a variety of positive and negative impacts on individual cognitive, motivational, emotional, social, and other skills. For example, systematic reviews and meta-analytic studies of existing research have found that exergames work as effectively as conventional exercise in reducing anxiety [18], and commercial games show promise to ameliorate the symptoms of a variety of mental conditions and may produce positive effects on players’ overall mental health [19]. On the opposing end, a meta-analysis of 101 studies focusing on children and adolescents found that playing video games is related to increased aggression and antisocial behavior as well as reduced academic performance [20]. With regard to mental well-being, one longitudinal study over a 3-week period in 7 English-speaking countries produced little to no evidence on the impact of time spent playing games on adult players’ well-being [21], whereas a natural experiment involving Japanese console players extending over a 2-year period found a positive causal path linking gaming and mental well-being [22]. In particular, the interaction among individual, environmental, and gaming-related factors may result in problematic behavior often classified as gaming disorder [23], for which the prevalence rate was found to be higher in East Asian countries than other regions in the world [24].

Video games can provide ways of coping during difficult lifetime experiences and unusual times of personal difficulty [25]. The COVID-19 pandemic, especially during shelter-in-place lockdowns, created a circumstance of uncertainty and disruption. A systematic review of research spanning diverse demographics across different national settings during the COVID-19 pandemic or living through lockdowns indicated that gaming had a beneficial outcome in diminishing stress and anxiety in general but it had a detrimental effect on certain at-risk individuals [26]. Moreover, limited research evidence with people caught in lockdowns revealed that playing video games provided a compensatory response to the stressors of the COVID-19 pandemic and served as a viable coping mechanism as a short-term distraction [27,28]. However, the broad-strokes nature of the wording in the survey questions [27] and the small number of participants [28] in these studies left many important questions unanswered.

### Therapeutic Use of Video Games

The digital revolution spearheaded by the smartphone, information networks, and personal communication technologies opens up unprecedented opportunities to tackle both the size and scale of the global mental health crisis, ranging from self-monitoring and self-management to online health interventions [29]. As video games become a ubiquitous part of global popular culture and mass entertainment, mounting attention has been paid by researchers and practitioners alike to the various roles video games play in the care of mental health for people (especially youth) with certain conditions in clinical and other settings, such as the therapeutic treatment of children and adolescents with attention-deficit/hyperactivity disorder, autism, anxiety disorders, and older adults with cognitive impairment [30-32]. As a result, gaming for mental well-being has been a rather well-trodden area of academic research and practical explorations [33,34].

The pervasiveness and relatively low cost of video games make them a primary candidate for consideration and adoption in clinical use. Emerging (albeit limited) evidence-based studies have shown that game-based interventions are useful and effective in treating patients with depressive disorders [35-37], and playing games has shown promise in leading to a clinically measurable reduction in anxiety disorder symptoms [38-40]. On the other hand, a systematic review of existing research shows that there is not much conclusive evidence thus far demonstrating the effectiveness or cost-effectiveness of digital mental health interventions, including those using video games, in the treatment of anxiety and depression disorders [41,42]. This is clearly an area that calls for more rigorous scrutiny by the academic community. It is worth highlighting that most of the research efforts examining the mental health implications of using video games as an intervention have been focused on serious games and games specifically designed to meet the needs of certain psychological and mental conditions, and these were not commercially available to the public [36,37,43-45]. However, there is growing realization that off-the-shelf games, thanks to their immense popularity and pervasive reach, have untapped potential for preventive and therapeutic applications [46,47]. Meanwhile, research investigating the applicability and efficacy of commercial games for diverse therapeutic use in recent years has gained increasing traction [48-52]. This is especially relevant during public health crisis situations such as the COVID-19 pandemic, when gaming technology assumes multiple functions in reconfiguring people’s lives [28,53] and evidence-based assessment is pivotal for understanding the potential roles of video games as coping mechanisms for diverse populations and demographics.

### Research Questions and Objectives

A review of recent studies on the therapeutic use of video games revealed a striking dearth of research aimed at the college-age cohort who not only are susceptible to depression and anxiety disorders but also are the most likely consumers of video games [40]. The purpose of this research was to examine the interplay of general-purpose video game play (ie, commercial games) and the mental health risks of anxiety and depression symptoms during periods of stringent shelter-in-place lockdowns among a national sample of college students in China. Lockdowns marked moments of drastic disruption of daily routines and provided an unusual opportunity to study improvised coping through readily available video games, and in this case, the role of video games in moderating anxiety and depression symptoms among college students. Specifically, our research questions were posited to investigate the association between several explanatory variables such as sex, year in college, platform (online PC vs mobile), amount of gameplay, modality of play, type of games, and the outcome variables of the severity of depressive and generalized anxiety disorder symptoms in college students living under conditions of shelter-in-place lockdowns in China. Moreover, the research also sought to test if there was significant difference in symptoms of depressive and generalized anxiety disorders between college students who started to play games and those who did not use video games during lockdown periods.

## Methods

### Participants and Procedure

College students were recruited from 16 geographic regions encompassing 8 provincial areas (ie, Beijing, Shanghai, Fujian, Henan, Hebei, Hunan, Hubei, and Zhejiang) across China through the service of a professional survey company. Data collection took place in December 2021 and January 2022, during an extended period of elevated infections across multiple regions in China during the COVID-19 pandemic. Many local government authorities enforced stringent shelter-in-place mandates in residential areas with diagnosed cases of patients with COVID-19, with public transportations suspended and checkpoints added at neighborhood units to curb human movement. A purposive sample of currently registered college students from 36 campuses who were under lockdown mandates at the time were recruited to participate in the web-based survey. To exclusively target college students who were caught under concurrent shelter-in-place lockdown mandates, 3 layers of filter were adopted by the survey company: first, only regions under lockdown mandates were chosen; second, a confirmation was obtained from respective sampled universities that a lockdown was in place; third, students chosen to participate were asked to start their survey with answering the question “Are you currently under COVID-19 lockdowns,” and only these who answered “yes” were directed to continue the survey, which was completed via Wenjuanxing [54], China’s most popular online survey platform. Most of the participants completed the questions on their smartphones, while the rest finished on a PC. Participants were reached via mobile phone, WeChat (Tencent Holdings Limited) account, or email and were first briefed with the overall purpose of the study and were assured anonymity; filling out the survey was entirely voluntary, and students could terminate at any time. For the sake of comparison, data from both gamers and nongamers were collected, and 2818 valid responses (n=1396, 49.53% male participants vs n=1422, 50.46% female participants) were received after incomplete questionnaires were discarded.

The quantitative survey data were supplemented by in-depth interviews of 17 informants (9 female participants vs 8 male participants; 14 undergraduate vs 3 graduate students). For both the survey and in-depth interviews, question items were initially developed based on feedback from 2 informal web-based focus groups (6 and 7 participants each) we conducted with college students under lockdowns; the wording of these items was then distributed to a dozen college students for comments and suggestions. Interviewees were recruited from multiple campuses using a snowballing sampling approach through soliciting recommendations from friends of friends based on a predetermined set of demographic attributes. Only video game users from students who were living under conditions of shelter-in-place lockdowns during December 2021 and January 2022 were included. A set of 8 open-ended questions were followed in the interviews pertinent to the practices, rationale, and perceived benefits or outcomes of video game engagement during shelter-in-place lockdowns (Multimedia Appendix 1). The interviews, which typically took 30 to 40 minutes, were completed in January 2022 via WeChat, China’s most popular instant messaging social media app, through audio or text chatting. The qualitative data collection was terminated once our iterative processing of the interview data led us to conclude that saturation was reached [55]. The interviews were recorded and transcribed for subsequent analysis.

### Ethical Considerations

The study protocol was approved by the Social Sciences Ethics Committee of Minjiang University (MJU.CJC.20210011). Informed consent was solicited electronically before the web-based survey, and participants were provided with detailed information regarding the research objectives, procedures, potential risks, and benefits of the study. Students aged <18 years were excluded from the research. At the beginning of the survey, each participant was asked to read and sign an informed consent form before answering the survey questions; a similar process was followed for the virtual interviews. Participants could opt out at any time, and survey takers who completed the questionnaire received ¥10 RMB (approximately US $1.40) cash payment at the end. All data collected were deidentified when being merged into a single data corpus.

### Measures

Since our primary goal was to investigate the association between video game play and the scale of college students’ anxiety and depression symptoms, we used the 7-item Generalized Anxiety Disorder (GAD-7) questionnaire for the screening of anxiety [56,57] and the self-administered 9-item Patient Health Questionnaire (PHQ-9) for the assessment of individual depression [58,59]. The questionnaires were initially developed in primary care and have now been widely adopted as reliable and valid measures in diagnosing depression and anxiety disorders, respectively, in clinical settings. The Chinese versions we used in the survey have been validated among diverse Chinese patients as well as the general population for their validity and reliability [60-62]. The instrument demonstrated high reliability (internal consistency) for both the GAD-7 and the PHQ-9 items in our dataset: Cronbach α was 0.953 for anxiety and 0.932 for depression. We followed the cutoff points recommended by clinical diagnosis in both the West [56,57,63] and among Chinese demographics [60-62] to categorize generalized anxiety disorder into minimal (score 0-4), mild (score 5-9), moderate (score 10-14), and severe (score 15-21) levels. Video game play was measured using a set of questions about whether and, if yes, how often survey participants played mobile or online games, whether they thought playing games was effective in reducing anxiety or depression, the modality of play (be it single person vs multiperson), daily hours of game time, and the type of games they played the most. Demographic variables included year in college and sex (Multimedia Appendix 2).

### Statistical Analysis

Statistical analysis was performed using SPSS for Windows (version 29; IBM). Descriptive statistics were reported as percentages for nominal variables and as means and SDs for ordinal or ranking variables. Multiple linear regression analysis was run to determine whether demographic variables and gameplay patterns accounted for the outcome of anxiety and depression, respectively. Preliminary diagnostic analyses were performed to test the assumptions for multiple regression modeling using anxiety disorder and depressive symptoms as respective outcome variables. Low multilinearity and appropriate homoscedasticity were confirmed for each regression model. The criterion variable was the composite score of the GAD-7 items for the anxiety regression model and the cumulative score of the PHQ-9 items for depression. Before being included in the regression model, an omnibus test was performed on each explanatory variable using a 1-way ANOVA model on the respective outcomes of anxiety and depressive symptoms, with all tests approaching statistical significance at the *P*<.05 level. The explanatory (ie, independent) variables were year in college (dummy coded; first-year student=0), whether gameplay was perceived to be effective (dummy coded; female=0), how often one played mobile games, how often one played online games, how many hours one spent playing games every day, change in daily playtime (from nonlockdown to lockdown days), modality of play (dummy coded; play alone=0), and type of games one played the most (dummy coded; single player/console games=0). Following research findings that playing video games during the COVID-19 pandemic might have a detrimental impact on certain players [26], we were interested in examining if the amount of game time played an important moderating role and therefore conducted ANOVA tests to compare groupwise variations based on the severity of anxiety and depressive disorder symptoms among college students during lockdowns.

For the qualitative interview data, we followed the logic of grounded theory coding as explained by Charmaz [64] in a 2-step analytic procedure. In the initial phase of “open-ended coding,” 2 researchers independently traversed the data corpus to pinpoint semantic categories and thematic patterns, which were then cross-examined, compared, and crystallized into coherent topic summaries. The second phase of “focused coding” allowed us to relate the interview data to meaningful gameplay experiences and salient themes. This structured approach aligns closely with the codebook thematic analysis paradigm highlighted by Clarke and Braun [65]. In the initial stage of data familiarization, the 3 researchers first worked independently and next worked together to reach consensus in the deductive development of coding frames; the subsequent inductive (ie, structured) mapping of the whole interview data corpus was analyzed by following the established coding scheme.

## Results

### Demographic Data and Descriptive Statistics

Of the 2818 survey respondents, there were 2669 (94.5%) undergraduate students, while there were 149 (5.3%) graduate and other students. Percentagewise, the first-year student cohorts dominated at 39.3% (1108/2818) of the total, followed by second-year student (811/2818, 28.8%), third-year student (486/2818, 17.2%), and fourth-year student (264/2818, 9.4%) demographics. Participants were almost evenly distributed along sex lines (1422/2818, 49.5% male participants vs 1396/2818, 50.5% female participants). Most of the surveyed students (1894/2818, 67.2%) reported playing games amid the COVID-19 pandemic shelter-in-place lockdowns, and male students were much more likely than their female counterparts to opt to play games during lockdowns (1030/1422, 73.8% vs 864/1396, 60.8%; χ^2^_1_=54.21, *P*<.001). For those who played games during lockdowns, the typical tendency was to play mobile games (mean 2.89, SD 0.95) and online games (mean 2.58, SD 1.02) rather frequently. For the sake of disambiguation, mobile games in China refer to those games that are typically played on smartphones, and online games are those that are played on a computer or handheld device. On average, student gamers played 2 to 3 (mean 2.62, SD 1.36) hours per day, and the amount of game time noticeably increased during lockdown moments than on nonlockdown days (mean 3.70, SD 0.84 hours). It should be acknowledged here that our question concerning increase in time included both mobile and PC online games in a single measurement, and it did not make separate inquiries. As for modality, most students played with their friends, colleagues, and other people they knew (1241/1894, 65.5%), and the types of games they played the most were massively multiplayer online games (674/1894, 35.6%); combat, competitive, or sports games (517/1894, 27.3%); and casual, chess, or card games (425/1894, 22.4%). Detailed statistics on the survey responses are outlined in Table 1.

**Table 1:** Participant characteristics.

Variables		Played games			
		Yes (n=1894)		No (n=924)	
**Year in college, n (%)**					
	1		781 (41.2)		327 (35.4)
	2		576 (30.4)		235 (25.4)
	3		285 (15)		201 (21.8)
	4		165 (8.7)		99 (10.7)
	Graduate student and other		87 (4.6)		62 (6.7)
**Sex, n (%)**					
	Male		1030 (54.4)		366 (39.6)
	Female		864 (45.6)		558 (60.4)
**Symptoms, mean (SD)**					
	Anxiety (GAD-7^a^) composite score (0-21)		4.47 (4.38)		4.87 (4.26)
	Depression (PHQ-9^b^) composite score (0-27)		7 (5.46)		7.11 (5.25)
**During the lockdowns, do you feel like playing video games is effective in reducing anxiety and depression? n (%)**					
	Yes		1047 (55.3)		N/A^c^
	No		847 (44.7)		N/A
During the lockdowns, how often do you play mobile games? (1=rarely; 2=occasionally; 3=often; 4=everyday), mean (SD)		2.89 (0.95)		N/A	
During the lockdowns, how often do you play online games? (1=rarely; 2=occasionally; 3=often; 4=everyday), mean (SD)		2.58 (1.02)		N/A	
On an average day, approximately how many hours do you play games during the lockdowns? (1=0-1 h; 2=1-2 h; 3=2-3 h; 4=3-4 h; 5=4-5 h; 6=>5 h), mean (SD)		2.62 (1.36)		N/A	
Compared with nonlockdown days, is there any change in the amount of time you spend playing games during the lockdowns? (1=much less; 2=somewhat less; 3=About the same; 4=somewhat more; 5=much more), mean (SD)		3.70 (0.84)		N/A	
**During the COVID-19 pandemic lockdowns, what was the most common modality of your gameplay? n (%)**					
	Play alone		556 (29.4)		N/A
	Play with friends (people I know)		1241 (65.5)		N/A
	Play with strangers		97 (5.1)		N/A
**Type of games played the most during the lockdowns, n (%)**					
	Single-player or console games		187 (9.9)		N/A
	Massively multiplayer online		674 (35.6)		N/A
	Casual, chess, and card games		425 (22.4)		N/A
	Combat, sports, or competitive games		517 (27.3)		N/A
	Strategy games		91 (4.8)		N/A

^a^GAD-7: 7-item Generalized Anxiety Disorder.

^b^PHQ-9: 9-item Patient Health Questionnaire.

^c^N/A: not applicable.

### Population-Level Differences Between Gamers and Nongamers

On average, survey respondents reported mild symptoms of anxiety (mean 4.60, SD 4.34) and depression (mean 7.03, SD 5.39), although there was a wide-ranging variance for both, as reflected in the SDs. Among the 1125 students who acknowledged playing games, 622 (55.3%) said doing this was effective in helping them deal with anxiety and depression. At the aggregate level, because a higher proportion of male students opted to play games than their female counterparts (as reported in the Demographic Data and Descriptive Statistics section), we ran a univariate, general linear model procedure using gameplay (responses=yes vs no) as the independent variable and anxiety and depression symptom scores as the respective dependent variable, with sex as the covariate to control for its potential cofounding effect. A significant video game effect was established between gamers and nongamers with regard to their overall anxiety disorder symptoms (mean 4.47, SD 4.38 vs mean 4.88, SD 4.27; *F*_1,2_=6.21; *P*=.01; η^2^=0.003) but not their depressive symptoms (mean 7, SD 5.46 vs mean 7.11, SD 5.25; *F*_1,2_=0.11; *P*=.75; η^2^=0.000).

### Video Game Play and Anxiety

Table 2 reports the coefficients of explanatory variables on the composite anxiety score. Being a male or female gamer did not play a significant role in accounting for whether gaming was impactful in mitigating anxiety symptoms. Compared with a first-year student, a second-year student (β=0.05, *P*=.04), fourth-year student (β=0.12, *P*<.001), or graduate or other student (β=0.06, *P*=.02) playing video games was more likely to experience elevated anxiety during lockdowns. Playing more online games (β=0.07, *P*=.03) and spending more time gaming in terms of the total daily hours (β=0.08, *P*=.005) and increase in time (β=0.06, *P*=.045) during lockdowns were associated with heightened anxiety symptoms. Compared with gaming alone, playing more casual, chess, or card games (β=0.08, *P*=.04) was linked to higher anxiety. On the other hand, playing with friends (β=−0.05, *P*=.04) was significantly linked to alleviated anxiety scores, and self-perceived efficacy of gameplay (β=−0.10, *P*<.001) contributed significantly to a lowered level of anxiety.

**Table 2:** Multiple linear regression coefficients toward anxiety disorder symptoms (N=1894).

Demographics and game behaviors		Unstandardized coefficients B (SE)	Standardized β coefficients (95% CI)	*t* ratio		*P* value
Constant		1.47 (0.56)	—^a^ (0.37-2.57)	2.61	.009	
Sex^b^ (male)		−0.16 (0.21)	−0.02 (−0.58 to 0.26)	−0.77	.44	
**Year in college^c^**						
	2	0.49 (0.24)	0.05 (0.03-0.95)	2.07	.04	
	3	0.53 (0.30)	0.04 (−0.06 to 1.12)	1.78	.08	
	4	1.88 (0.37)	0.12 (1.16-2.60)	5.09	<.001	
	Graduate and other	1.19 (0.49)	0.06 (0.235-2.15)	2.44	.02	
EF^d,e^ (yes)		−0.85 (0.22)	−0.10 (−1.27 to −0.42)	−3.90	<.001	
MG^f^ (rating 1-4)		0.22 (0.13)	0.05 (−0.30 to 0.46)	1.72	.09	
OG^g^ (rating 1-4)		0.28 (0.13)	0.07 (0.03-0.53)	2.23	.03	
HR^h^ (rating 1-6)		0.26 (0.09)	0.08 (0.08-0.44)	2.79	.005	
CH^i^ (rating 1-5)		0.26 (0.14)	0.06 (−0.04 to 0.52)	1.85	.04	
**MD^j,k^**						
	With friends	−0.49 (0.23)	−0.05 (−0.94 to −0.02)	−2.08	.04	
	With strangers	−0.60 (0.48)	−0.03 (−1.54 to 0.34)	−1.25	.21	
**TE^l,m^**						
	Massively multiplayer online	0.47 (0.37)	0.05 (−0.25 to 1.19)	1.29	.20	
	Casual, chess**,** or card games	0.79 (0.38)	0.08 (0.05-1.54)	2.10	.04	
	Combat or sports	0.42 (0.38)	0.04 (−0.32 to 1.15)	1.10	.30	
	Strategy	0.63 (0.55)	0.03 (−0.45 to 1.72)	1.14	.25	

^a^Not available.

^b^Dummy coding; female=0.

^c^Dummy coding; first-year student=0.

^d^EF: during the lockdowns, do you feel like playing video games is effective in reducing anxiety and depression?

^e^Dummy coding; effective (no)=0.

^f^MG: during the lockdowns, how often do you play mobile games?

^g^OG: during the lockdowns, how often do you play online games?

^h^HR: on an average day, approximately how many hours do you play games during the lockdowns?

^i^CH: compared with nonlockdown days, is there any change in the amount of time you spend playing games during the lockdowns?

^j^MD: during the COVID-19 pandemic lockdowns, what was the most common modality of your gameplay?

^k^Dummy coding; play alone=0.

^l^TE: type of games played the most during the lockdowns.

^m^Dummy coding: single-player or console games=0.

### Video Game Play and Depression

Regarding depressive disorder symptoms (Table 3), no significant sex effect was detected, whereas being a second-year student (vs a first-year student; β=0.07, *P*=.003), third-year student (β=0.05, *P*=.03), or fourth-year student (β=0.09, *P*<.001) increased the likelihood of feeling depressed. Similar to the anxiety regression model, increased online gameplay (β=0.07, *P*=.02) and more playtime as measured in the total daily hours (β=0.07, *P*=.01) and surge in time (β=0.06, *P*=.02) were significantly related to increases in depression levels. Conversely, social play with friends (β=−0.08, *P*=.003) was a significant contributor to reduced depressive disorder. As with the anxiety model, self-perception of video game efficacy (β=−0.08, *P*<.001) significantly accounted for mitigated depressive symptoms.

**Table 3:** Multiple linear regression coefficients toward depression disorder symptoms (N=1894).

Demographics and game behaviors		Unstandardized coefficients, B (SE)		Standardized β coefficients (95% CI)		t-ratio		*P* value	
Constant		3.43 (0.70)		—^a^ (2.06-4.81)		4.90		<.001	
Sex^b^ (male)		−0.41 (0.27)		−0.04 (−0.93 to 0.11)		−1.55		.12	
**Year in college^c^**									
	2		0.89 (0.30)		0.07 (0.31-1.47)		3.01		.003
	3		0.79 (0.37)		0.05 (0.06-1.52)		2.12		.03
	4		1.75 (0.46)		0.09 (0.84-2.65)		3.78		<.001
	Graduate and other		0.60 (0.61)		0.02 (−0.60-1.79)		0.97		.33
EF^d,e^ (yes)		−0.92 (0.27)		−0.08 (−1.46 to −0.39)		−3.41		<.001	
MG^f^ (1-4)		0.28 (0.16)		0.05 (−0.02 to 0.59)		1.81		.07	
OG^g^ (1-4)		0.36 (0.16)		0.07 (0.05-0.67)		2.28		.02	
HR^h^ (1-6)		0.30 (0.12)		0.07 (0.07-0.52)		2.54		.01	
CH^i^ (1-5)		0.41 (0.18)		0.06 (0.07-0.76)		2.33		.02	
**MD^j,k^**									
	With friends		−0.87 (0.29)		−0.08 (−1.44 to −0.30)		−2.99		.003
	With strangers		−0.39 (0.60)		−0.02 (−1.56 to 0.79)		−0.64		.52
**TE^l,m^**									
	Massively multiplayer online		0.16 (0.46)		0.01 (−0.74 to 1.06)		0.34		.73
	Casual, chess, or card games		0.55 (0.47)		0.04 (−0.38 to 1.48)		1.17		.24
	Combat or sports		0.25 (0.47)		0.02 (−0.68 to 1.17)		0.52		.60
	Strategy		0.35 (0.69)		0.01 (−1.01 to 1.71)		0.51		.61

^a^Not available.

^b^Dummy coding; female=0.

^c^Dummy coding; first-year student=0.

^d^EF: during the lockdowns, do you feel like playing video games is effective in reducing anxiety and depression?

^e^Dummy coding; effective (no)=0.

^f^MG: during the lockdowns, how often do you play mobile games?

^g^OG: during the lockdowns, how often do you play online games?

^h^HR: on an average day, approximately how many hours do you play games during the lockdowns?

^i^CH: compared with nonlockdown days, is there any change in the amount of time you spend playing games during the lockdowns?

^j^MD: during the COVID-19 pandemic lockdowns, what was the most common modality of your gameplay?

^k^Dummy coding; play alone=0.

^l^TE: type of games played the most during the lockdowns.

^m^Dummy coding: single-player or console games=0.

### Association Between Video Game Play and Severity of Anxiety and Depression

In terms of severity levels in gamer versus nongamer (n=924) distribution, the prevalence rate was 51.53% (n=976) versus 48.38% (n=447) for the minimal level, 38.17% (n=723) versus 39.83% (n=368) for the mild level, 8.39% (n=159) versus 9.85% (n=91) for the moderate level, and 1.9% versus 1.95% (n=18) for the severe level for anxiety symptoms. Similarly, following standard clinical practice [58,59,66], total scores of <5, 5, 10, 15, and 20 were used as cutoff points for the minimal, mild, moderate, moderately severe, and severe levels of depression, respectively. Comparing gamers with nongamers (n=924), the prevalence rate for depressive symptoms was 35.01% (n=663) versus 33.98% (n=314) for the minimal level, 41.55% (n=787) versus 43.40% (n=401) for the mild level, 13.41% (n=254) versus 14.50% (n=134) for the moderate level, 7.39% (n=140) versus 5.52% (n=51) for the moderately severe level, and 2.64% (n=50) versus 2.6% (n=24) for the severe level.

As the number of daily hours of gametime and the degree of change in playtime from before the lockdown to during the lockdown in the COVID-19 pandemic were 2 prominent intervening factors influencing video game use on one hand and both anxiety and depressive disorders on the other hand, we further analyzed the respective role of these 2 factors in modulating the levels of anxiety and depressive symptoms.

A 1-way ANOVA test using daily playtime as the dependent variable and anxiety severity as the grouping variable indicated significant groupwise differences (*F*_3,1890_=14.10; *P*<.001; η^2^=0.022; 95% CI 0.011-0.036). As outlined in Table 4, students with severe anxiety and moderate anxiety departed significantly from all other cohorts in their daily playtime, with pairwise 2-tailed *t* tests displaying statistical significance at the *P*<.01 level. In terms of prelockdown to lockdown playtime change during the COVID-19 pandemic, it can be seen from Table 5 that students with severe anxiety symptoms differed significantly from all other groups (*F*_3,1890_=9.29; *P*<.001; η^2^=0.016; 95% CI 0.006-0.028). In other words, students experiencing severe anxiety symptoms also registered the biggest increase in daily gametime (*P*<.001 against all other cohorts).

**Table 4:** Pairwise comparisons and Cohen d values for the association between the amount of daily playtime^a^ and anxiety severity^b^.

		Mean difference (95% CI)	Cohen *d*	*t* test^c^ (*df*)	*P* value
**Severe anxiety versus...**					
	Moderate anxiety	0.78 (0.24-1.32)	0.53	2.87 (193)	.005
	Mild anxiety	1.21 (0.76-1.66)	0.90	5.26 (757)	<.001
	Minimal anxiety	1.22 (0.78-1.67)	0.91	5.36 (1010)	<.001
**Moderate anxiety versus...**					
	Mild anxiety	0.43 (0.19-0.66)	0.32	3.61 (880)	<.001
	Minimal anxiety	0.44 (0.21-0.67)	0.33	3.82 (1133)	<.001
Mild anxiety versus minimal anxiety		0.01 (−0.11 to 0.14)	0.01	0.21 (1697)	.83

^a^Daily playtime is measured using a 1 to 6 scale. Table 1 provides further details (referenced as HR).

^b^Descriptive statistics of daily playtime amount per anxiety severity levels: severe anxiety (n=36; mean 3.78, SD 1.66); moderate anxiety (n=159; mean 2.99, SD 1.44); mild anxiety (n=723; mean 2.57, SD 1.33); and minimal anxiety (n=976; mean 2.55, SD 1.33).

^c^All *t* tests were 2-tailed.

**Table 5:** Pairwise comparisons and Cohen d values for the association between the change in daily playtime^a^ and anxiety severity^b^.

			Mean difference (95% CI)		Cohen *d*		*t* test^c^ (*df*)		*P* value
**Severe anxiety versus...**									
	Moderate anxiety	0.59 (0.27 to 0.92)		0.67		3.62 (193)		<.001	
	Mild anxiety	0.63 (0.36 to 0.90)		0.79		4.61 (757)		<.001	
	Minimal anxiety	0.71 (0.43 to 0.99)		0.85		5.01 (1010)		<.001	
**Moderate anxiety versus...**									
	Mild anxiety	0.04 (−0.10 to 0.18)		0.05		0.53 (880)		.60	
	Minimal anxiety	0.12 (−0.03 to 0.26)		0.14		1.59 (1133)		.11	
Mild anxiety versus minimal anxiety			0.08 (−0.002 to 0.16)		0.09		1.91 (1697)		.06

^a^Change in daily playtime is measured using a 1 to 5 scale. Table 1 provides further details (referenced as CH).

^b^Descriptive statistics of change in daily playtime per anxiety severity levels: severe anxiety (n=36; mean 4.36, SD 0.72); moderate anxiety (n=159; mean 3.77, SD 0.92); mild anxiety (n=723; mean 3.73, SD 0.81); and minimal anxiety (n=976; mean 3.66; SD 0.84).

^c^All *t* tests were 2-tailed.

A 1-way ANOVA test identified a significant effect of daily game time on the level of depression severity (*F*_4,1889_=9.79; *P*<.001; η^2^=0.021; 95% CI 0.009-0.035). Post hoc pairwise tests pinpointed the severe depressive cohort as deviating from all other groups (*P*<.01), as reported in Table 6. While the 1-way ANOVA omnibus test showed overall significance with regard to change in playtime (*F*_4,1889_=7.03; *P*<.001; η^2^=0.016; 95% CI 0.006-0.026), post hoc groupwise comparisons again pointed to the severe depressive group being consistently distanced from all other groups (*P*<.001; Table 7).

**Table 6:** Pairwise comparisons and Cohen d values for the association between the amount of daily playtime^a^ and depression severity^b^.

		Mean difference (95% CI)		Cohen *d*		*t* test^c^ (*df*)			*P* value
**Severe depression versus...**									
	Moderately severe depression		0.68 (0.17-1.19)		0.44		2.64 (188)	.009	
	Moderate depression		0.89 (0.46-1.31)		0.64		4.11 (302)	<.001	
	Mild depression		0.98 (0.60-1.36)		0.73		5.04 (835)	<.001	
	Minimal depression		1.10 (0.70-1.49)		0.80		5.43 (711)	<.001	
**Moderately severe depression versus...**									
	Moderate depression		0.21 (−0.08 to 0.49)		0.15		1.43 (392)	.16	
	Mild depression		0.30 (0.06 to 0.54)		0.23		2.46 (925)	.01	
	Minimal depression		0.42 (0.17 to 0.67)		0.30		3.27 (801)	.001	
**Moderate depression versus...**									
	Mild depression		0.09 (−0.09 to 0.28)		0.07		1.00 (1039)	.32	
	Minimal anxiety		0.21 (0.02-0.40)		0.16		2.13 (915)	.03	
Mild depression versus minimal depression		0.12 (−0.02 to 0.25)		0.09		1.66 (1448)			.10

^a^Daily playtime is measured using a 1 to 6 scale. Table 1 provides further details (referenced as HR).

^b^Descriptive statistics of daily playtime amount per depression severity levels: severe depression (n=50; mean 3.58, SD 1.75); moderately severe depression (n=140; mean 2.90, SD 1.49); moderate depression (n=254; mean 2.69, SD 1.32); mild depression (n=787; mean 2.60, SD 1.31); and minimal depression (n=663; mean 2.48, SD 1.35).

^c^All *t* tests were 2-tailed.

**Table 7:** Pairwise comparisons and Cohen d for the association between change in daily playtime^a^ and depression severity^b^.

		Mean difference (95% CI)		Cohen *d*		*t* test^c^ (*df*)		*P* value	
**Severe depression versus...**									
	Moderately severe depression		0.38 (0.10-0.66)		0.44		2.70 (188)		.008
	Moderate depression		0.42 (0.16-0.67)		0.50		3.25 (302)		.001
	Mild depression		0.51 (0.28-0.75)		0.62		4.25 (835)		<.001
	Minimal depression		0.57 (0.32-0.81)		0.67		4.58 (711)		<.001
**Moderately severe depression versus...**									
	Moderate depression		0.04 (−0.13 to 0.21)		0.05		0.44 (392)		.66
	Mild depression		0.13 (−0.01 to 0.28)		0.16		1.77 (925)		.08
	Minimal depression		0.19 (0.04-0.34)		0.23		2.42 (801)		.02
**Moderate depression versus...**									
	Mild depression		0.10 (−0.02 to 0.21)		0.12		1.62 (1039)		.11
	Minimal anxiety		0.15 (0.03-0.27)		0.18		2.46 (915)		.01
Mild depression versus minimal depression		0.06 (−0.03 to 0.14)		0.07		1.26 (1448)		.21	

^a^Change in daily playtime is measured using a 1 to 5 scale. Table 1 provides further details (referenced as CH).

^b^Descriptive statistics of change in daily playtime per depression severity levels: severe depression (n=50; mean 4.20; SD=0.88); moderately severe depression (n=140; M=3.82; SD 0.84); moderate depression (n=254; mean 3.78, SD 0.82); mild depression (n=787; mean 3.69, SD 0.82); minimal depression (n=663; mean 3.63, SD 0.84).

^c^All *t* tests were 2-tailed.

### Thematic Patterns of Qualitative Interview Data

An analysis of our in-depth interview data revealed several salient themes in the context of game use under COVID-19 pandemic lockdowns. First, the interviewees consistently pointed out a few circumstantial factors leading them to gravitate toward more gameplay during shelter-in-place moments: excessive exposure to the computer monitor and other digital devices where video games are easily available (15/17, 88%); suddenly gained extra hours that could lead to boredom if not occupied (13/17, 76%); escapism found in video games that could offer a temporary mental release from the grim realities of the COVID-19 pandemic and the lockdown (12/17, 71%); and isolation due to blockage of mobility that boosted the urge for sociality (11/17, 65%). Besides its entertainment value, gaming was cited by about one-third (7/17, 41%) of the interviewees as a preferred way to get together with friends and colleagues to chat while playing. Playing with family members was mentioned by some (5/17, 29%) to be an effective means in creating distractions from the lockdowns and in collectively coping with the challenging living conditions through bonding and emotional support. This overall sentiment is aptly illustrated by a quote from a graduate student interviewee:

Playing videogames gives me the opportunity to hook up with my good buddies that I otherwise would not be able to do during normal days when everybody is busy. I know my friends also play games, and we would first talk to one another on social media and find a common time. Once we are in the game, we turn the audio chat feature on and talk during the whole gameplay. It is a great way to catch up with one another and release myself emotionally.

Participant A, female participant, second year of doctoral studies

Besides socializing with friends, video game was also mentioned by a few interviewees as serving the role of a virtual companion, as imparted in this quote from a male third-year student participant:

Having the game on is a way to have a company with me, even when I am doing other things by myself...I use the auto-play calling feature to let NBA2K play on my computer. It is like a spiritual pal sitting by me.

Participant B, male participant, third year of doctoral studies

While a slight majority of the informants (10/17, 59%) indicated playing games helped them in handling their anxiety and pressure, others acknowledged its complicated nature as a way of coping with mental well-being. Some interviewees mentioned the transient nature of the euphoric feelings they obtained in the game-on environment, as demonstrated from this quote:

At times of boredom, playing games fills the void and brings joy to me. But after the gameplay, my downcast feeling kicks back in when the reality of COVID-19 and the lockdown returns to mind.

Participant C, female participant, third-year student undergraduate student

Another third-year college student described her game experience as bringing her “fleeting indulgence but durable angst” while living in the shadows of the lockdown. Moreover, 3 interviewees acknowledged that their bad mood during lockdowns often spilled over to gameplay, resulting in verbal confrontations, cursing, and other conflicts with other players. Playing under these conditions sometimes could further exacerbate their mental state. Out of 17 interviewees, 4 (24%) highlighted the double-edged nature of gaming during lockdowns: always being close to the computer, coupled with excessive leisure time, created the hard-to-resist temptation to enter the game world and stay there more than they wanted. In those cases, the joy of gaming was acknowledged to be transient, but this habitual behavior could eventually elevate one’s anxiety and depressive symptoms.

## Discussion

### Principal Findings

First, we would like to foreground a discussion of the particularity of the environmental setting of the COVID-19 pandemic and its lockdowns, which is pivotal for extrapolating our findings to other contexts. The prolonged COVID-19 pandemic disrupted routine habits and behaviors and has taken a profound toll on mental well-being. Stay-at-home lockdowns were adopted in most countries in the early stages of the COVID-19 pandemic as a measure to hinder the spread of the virus. However, China’s zero-COVID policy and its enforcement differentiated the Chinese shelter-in-place lockdowns from those in the rest of the world due to the extent of closures and reduction in human mobility and contact. The draconian nature of the COVID-19 pandemic lockdown measures triggered widespread protests (aka the “A4 Blank Paper Movement”) in 21 provinces and over 200 college campuses in late 2022, which led to the end of the regime’s zero-COVID policy [67]. It is useful to take the environmental circumstances of the Chinese lockdown into consideration while evaluating the findings and the analysis.

Research in cross-national settings has found that video games are helpful in reducing stress, anxiety, and depression among college students and other demographics during the stay-at-home period following the COVID-19 outbreak [26,28,53]. Findings in this research showed that, in comparison with nonplayers, video game players were linked to overall lower anxiety symptoms but not depressive disorder symptoms at the level of the study population, controlling for the effect of sex. Because our research design was cross-sectional and correlational in nature, we cannot draw conclusions on causation concerning the efficacy of video games based on the regression models. Nonetheless, ancillary evidence in support of the benefits of video games in mitigating anxiety and depression came from the survey responses directed to the question asking the informants whether playing video games produced such effects; 55.3% of those who played games answered in the affirmative. The same pattern was corroborated in the in-depth interviews. However, it must be pointed out that several pivotal individual and environmental factors that were absent from this research are needed for a full understanding of the behavioral tendencies and their associated mental health outcomes. For example, physical exercise, social connections and support, living conditions, and previous gaming experience could play an important moderating role in the process. In this regard, control groups and longitudinal data would be most valuable in dissecting the specific role video games play in coping with anxiety and depression under stringent life moments.

A sizable proportion of the surveyed students did not perceive video games to be effective in coping with anxiety and depression. This probably comes as no surprise, because gaming-related behavior could result in counterproductive and detrimental consequences in a certain segment of players, especially problematic ones, at moments of uncertainty and disruption such as the COVID-19 pandemic and in the face of drastic measures of the lockdowns [26]. Longitudinal data show that avoidant coping (tendency to retreat or divert attention from the pandemic) and excessive gaming time (which happened at the expense of social networking time and perceived family support) were associated with greater distress during the extended COVID-19 pandemic stay-at-home period [68]. Our study reveals that college students who spent an extended amount of time playing video games were associated with severe levels of anxiety and depression, and students in the severe anxiety and depression cohorts were also the most likely to increase gaming time during shelter-in-place lockdowns. This pattern is highly congruent with findings in the extant research literature in this area [26]. This suggests that certain at-risk individuals who are susceptible to gaming disorders should be closely monitored at difficult times, as coping with video games has the potential to aggravate, not mitigate, the mental health state.

The role of video games goes beyond a mere entertainment product, as playing with one’s social circle of friends, colleagues, and other close connections facilitates casual catch-ups and informal chitchats, which in turn would aid in dealing with personal anxiety and depression challenges. Self-determination and modality of play are important factors in determining outcomes [27,69]. Findings presented in this research show that social playing (with family and friends) was significantly correlated with both reduced generalized anxiety and depressive disorder symptoms. This is corroborated with evidence from the in-depth interviews, as many participants indicated that simply connecting with friends and intimate circles in the game sphere had the effects of helping them feel connected and distracted from on-hand difficulties and distress. Loneliness, which was exacerbated by social isolation, was found to be strongly linked to anxiety and depressive symptoms during the COVID-19 outbreak [70]. Meanwhile, social motivations for gaming could potentially have the benefits of reducing loneliness, but they could accentuate gaming disorders and therefore negatively impact mental health [71]. However, the lack of survey tools in measuring diverse motivational factors from this study limits our perspectives in further understanding these dynamics. Nonetheless, the complex relationships and interplays of these pivotal factors will be worthy of further exploration under diverse circumstances.

Although the questionnaire used in this study did not measure internet gaming disorder, an accumulated body of empirical evidence indicates that the primary predictor of this disorder rests with excessive game time, and gaming disorder shows a high comorbidity with depressive and anxiety disorders [72,73]. On the basis of triangulating evidence from diverse settings, there is reason to speculate that the buffering effects of videogaming may be the most asserting among routine players with moderate to low anxiety and depression symptoms, while this impact is compounded by disorderly gaming behaviors and may incur adverse consequences on those who are already struggling with high anxiety and depression [26,49,69,72,73]. Compared with other types of players, only problematic gamers among Chinese adolescents showed a positive association with anxiety severity [74]. Other research has indicated that students experiencing life stressors display fewer depressive symptoms while using adaptive coping strategies compared with maladaptive ones [75-77]. The divergent paths of adaptive (ie, proactive) and maladaptive (ie, reactive or avoidant) coping styles including the use of video games toward outcomes of alleviating anxiety and depression should warrant further scrutiny from researchers and practitioners alike. This points to the important role of intentionality in modulating the impact of video game adoption during times of unforeseen, heightened mental distress and uneasiness, which may offer useful guidance for college counselors and advisers as well as parents and college students themselves in favor of adaptive (ie, proactive) rather than maladaptive (ie, reactive) strategies in using video games to cope with excruciating and unanticipated stressors. Moreover, the comorbid condition of gaming disorder with anxiety and depression disorder symptoms among certain at-risk individuals remains a vital area of empirical scrutiny.

### Promising Directions for Future Research

Gameplay modality and game content are important moderating factors to contemplate in examining the role of gaming as an intervention for anxiety and depression. The socializing aspects of video games have been claimed to boast great potential in accruing social support and rapport and thus boost well-being [33,78], with limited support from emerging research on video game use during the COVID-19 pandemic [26]. Our research pinpointed a prominent association of social playing (ie, with friends, colleagues, and people one knows) with lowered anxiety and depressive symptoms, corroborated in both the quantitative survey and the qualitative interviews. Systematic and scoping reviews of extant research investigating the effects of video games on anxiety and depression have consistently detected a surprising paucity of focus on this particular (ie, socializing) aspects of gameplay [30,31,34,37,39,41,43,48,50,69], and this is a fruitful area of future research for both theory testing and practical guidance. Regarding game content, evidence is inconclusive from our survey to interpret the role of engagement with specific game types. This may be more of an issue of a lack of precise or appropriate classification and typology in our study and calls for improvement in future research.

### Limitations

Several limitations should be acknowledged surrounding the design and accomplishment of this research. The use of self-reported data might involve inaccuracies in important measurements, which formed the basis of our analysis. Selection bias in both the survey and in-depth interview participants could have tainted the results in certain directions. The survey tools only focused on some aspects and did not cover many potentially influential factors at the individual, social, and ecological levels that could have played essential roles in determining video game effects. In addition, preexisting mental health conditions and previous habitual video game experience may be pivotal in moderating engagement outcomes at moments of public health crises such as the COVID-19 outbreak. Therefore, diverse and expanded assessment tools on pivotal parameters from symptom diagnosis to individual traits and behavioral tendencies are desired. The single-shot, cross-sectional, and correlational design creates challenges for causal inferences and generalizations. There was a high level of fluidity and unpredictability throughout the COVID-19 outbreak, and lockdown policies and measures showed intense variations from region to region as well as from country to country. Constrained by the particular timing of the survey, the results reported in the study could only shed light on the short-term impact and would not be able to address long-term ramifications. The survey was conducted at a particular moment in China’s zero-COVID crusade across multiple geographic regions, which might have introduced peculiar environmental conditions and unusual gameplay dynamics hard to account for in the data and the analysis. As a result, sequential or causal inferences cannot be drawn between video game use and mental health outcomes as related to anxiety and depression among the respondents; nor can the findings be easily generalizable to the large population. Nonetheless, the findings in this study offer much-needed insight on empirical evidence on the interplay of different modalities of video game play and various manifestations of generalized anxiety and depressive disorders at an unusual public health crisis moment.

### Conclusions

This study investigated college students’ video game play during the draconian COVID-19 shelter-in-place lockdowns in China. Overall, compared with nonuse, playing video games during lockdowns was linked to significantly lowered anxiety symptoms but not sizable differences in depressive disorder symptoms. There were nuanced variations at the individual level with regard to those who resorted to playing video games as a way to deal with mental well-being. First, it is worth highlighting that the self-perceived efficacy of video games in mitigating the downsides of anxiety and depressive disorders served as a significant predictor for lowered symptoms. Playing video games did not affect college students at all stages, as second-year students and fourth-year students were much more likely to have both anxiety and depression than their counterparts, when game playing was held constant. Compared with mobile games, online games were more effective at alleviating anxiety and depressive symptoms. Playing in moderation (ie, for a modest amount of time) was found to be positively associated with reduced anxiety and depressive symptoms. Conversely, excessive engagement with gaming and a dramatic increase in playtime during lockdowns were linked to high levels of anxiety and depressive disorders. Social play with friends and family members (but not strangers) was positively correlated with alleviated anxiety and depression scores. These findings could offer actionable and pragmatic tips for guidance with college counselors and mental health professionals in their effort to help students cope with turmoil and disruption.

## Data Availability

The datasets generated and analyzed in this study are available from the corresponding author upon reasonable request.

## Appendix

Multimedia Appendix 1 In-depth interview guide.

Multimedia Appendix 2 Wenjuanxing online survey questionnaire.

## Abbreviations

GAD-7: 7-item Generalized Anxiety Disorder
PHQ-9: 9-item Patient Health Questionnaire
