# Supplementary material for: The Positive Effect of Video-Game Play on College Students’ Anxiety and Depression Symptoms During the COVID-19 Pandemic Shelter-in-Place Lockdowns: Mixed Methods Study
Source: JMIR Serious Games. 2025 May 30;13:e58857. doi: 10.2196/58857 (PMC12166324; doi:10.2196/58857)
Supplement: Multimedia Appendix 1 [file games_v13i1e58857_app1.docx]

**深度访谈问卷**

首先，说明本次访谈的目的，以及邀请学生参加这次访谈，感谢他们的积极参与。访谈参加完全自愿，回答本身无对错之分，请他们如实回答。请确认访谈对象正处居家隔离期间，并讲采访对象限于玩游戏的同学。参加访谈的同学身份保密，数据经过非个性化后仅作研究只用。

**以下为访谈主要问题。如有需要，请问相关后续问题。**

1. 性别，年级，所在学校和专业
2. 疫情居家期间，您玩游戏的行为同疫情之前相比是否有所变化（时间、游戏内容、游戏玩法等）？
3. 居家隔离期间，网游和手游，您是否都有玩过？哪一类游戏花的时间更多些？
4. 您游戏的主要方式，是主要一个人玩还是同别人一起玩居多？哪一种方式对于降低精神压力或者解除烦躁和心理忧虑更有帮助？
5. 居家隔离期间，您觉得玩游戏对于降低精神压力，是否有缓解作用？能否解释一下。
6. 居家隔离的日子里，游戏对于解除烦躁和心理忧虑是否有效？请仔细说明。
7. 疫情居家期间，家人或朋友对你玩游戏的态度和看法是否有所变化？
8. 就居家隔离期间玩游戏的总体体验，您有哪些评价？对于您本人来说，正面的作用有哪些？负面的作用有哪些？
