# Supplementary material for: The Positive Effect of Video-Game Play on College Students’ Anxiety and Depression Symptoms During the COVID-19 Pandemic Shelter-in-Place Lockdowns: Mixed Methods Study
Source: JMIR Serious Games. 2025 May 30;13:e58857. doi: 10.2196/58857 (PMC12166324; doi:10.2196/58857)
Supplement: Multimedia Appendix 2 [file games_v13i1e58857_app2.docx]

**提示与说明**

非常感谢您参与这次问卷调查。我们本研究目的是调查新冠疫情居家隔离期间大学生心理状态及处理应对方式。请您根据实际情况如实回答相关问题。

**您的年龄**（截至2021年12月）______ 周岁

**您所在的年级**：1. 大学一年级；2. 大学二年级；3. 大学三年级；4. 大学四年级；5. 硕士研究生；6. 博士研究生 7. 其他

**隔离期间您是在城市还是在农村？ 1. 城市 2. 城郊 3. 农村**

**所在大学 ________ 所学专业**： ____________

**性别**： 1. 男 2. 女

**疫情居家隔离期间，您的居住状况是：**

1. 同父母居住
2. 同父母以外的家人居住
3. 自己单独居住
4. 同朋友居住
5. 其他

**疫情隔离期间，你所在小区或者村庄的管控措施：**

1. 进出比较松散
2. 进出管控一般
3. 进出管控非常严格

**您是否有朋友或家人被确诊新冠？**1. 是 2. 否

**您所在小区是否有人被确诊新冠？**1. 是 2. 否

**新冠疫情隔离期间，有多少时候您受到以下情况的困扰？（每一项问题请选择一个答案）**

Q1、**做事情时提不起劲或少有乐趣**

 完全不会

 时常

 一半以上的日子

 几乎每天

Q2、**感到心情低落、沮丧或绝望**

 完全不会

 时常

 一半以上的日子

 几乎每天

Q3、**入睡困难、很难熟睡，或者睡觉太多**

 完全不会

 时常

 一半以上的日子

 几乎每天

Q4、**感觉疲劳或无精打采**

 完全不会

 时常

 一半以上的日子

 几乎每天

Q5、**胃口不好或吃东西太多**

 完全不会

 时常

 一半以上的日子

 几乎每天

Q6、**觉得自己状态很糟，或很失败，让自己或家人失望**

 完全不会

 时常

 一半以上的日子

 几乎每天

Q7、**很难集中精神于事物，或者难以专注做事**

 完全不会

 时常

 一半以上的日子

 几乎每天

Q8、**您烦躁或坐立不安情况比平常更严重，或正好相反 - 动作或说话速度比平常明显缓慢？**

 完全不会

 时常

 一半以上的日子

 几乎每天

Q9、**有厌世或用某种方式伤害自己的念头**

 完全不会

 时常

 一半以上的日子

 几乎每天

**隔离期间您在上网课吗？**1. 是 2. 否

**疫情隔离期间，请您回答是否存在下列描述的状况及频率，请选择符合您的选项**

Q1. **感觉心神不安、焦虑或高度紧张**

几乎没有

时常有

刚超过一半天数

几乎每天

**Q2. 不能停止或控制担心**

几乎没有

时常有

刚超过一半天数

几乎每天

**Q3. 为各种各样的事情过度担心**

几乎没有

时常有

刚超过一半天数

几乎每天

**Q4. 难以放松自己**

几乎没有

时常有

刚超过一半天数

几乎每天

**Q5. 非常不安静以至于难以坐定**

几乎没有

时常有

刚超过一半天数

几乎每天

**Q6. 变得易恼火或易急躁**

几乎没有

时常有

刚超过一半天数

几乎每天

**Q7. 害怕可怕的事情会发生**

几乎没有

时常有

刚超过一半天数

几乎每天

**新冠疫情隔离期间， 为了降低精神压力，解除烦躁和忧虑，您觉得哪些活动和方式最为有效？（请选择4-5项）**

各项健身活动

跟同学、朋友之间交流

听音乐

专心学业、钻研学问

同父母、家人交流

看网剧

下厨房学做美食

刷短视频

玩电子游戏

看小说等非专业书籍

看电视节目

睡懒觉

饲养宠物

玩社交媒体

玩电子游戏

**您在新冠以前是否患过焦虑症或抑郁症？**1. 有 2. 没有

**新冠疫情隔离期间，您是否会玩手游或者网络游戏?**

1. 有（请继续回答以下问题） 2. 没有 (跳至问卷结束)

**新冠疫情隔离期间, 你玩手游的频率：**

1. 很少玩

3. 偶尔玩

4. 经常玩

5. 有几乎天天玩

**新冠疫情隔离期间, 你玩网游的频率：**

1. 很少玩

3. 偶尔玩

4. 经常玩

5. 有几乎天天玩

**居家隔离期间，一般来说，您一天大概花多少时间玩游戏：**

0-1小时

1-2小时

2-3小时

3-4 小时

4-5 小时

5-6小时

6小时以上

**居家隔离期间，您玩游戏的时间：**

比平常少得多

比平常少一些

跟平常差不多

比平常多一些

比平常多出很多

**居家隔离期间，您玩游戏方式最多的是：**

自己一个人单独玩

同熟悉的朋友通过网络一起玩

同不熟悉的玩家一起玩

**居家隔离期间，您玩得最多的是一些哪一类的游戏？**

多人在线角色类

休闲类

棋牌类

对战类

竞技、体育类

策略类

单机其他

**最后，请您列举疫情隔离期间玩得最多的2-3部游戏名称： ________**

**问卷结束。我和我的研究团队非常感谢您的参与！**
